# Malignant atrophic papulosis (Köhlmeier-Degos disease) - A review

**DOI:** 10.1186/1750-1172-8-10

**Published:** 2013-01-14

**Authors:** Athanasios Theodoridis, Evgenia Makrantonaki, Christos C Zouboulis

**Affiliations:** 1 Departments of Dermatology, Venerology, Allergology and Immunology, Dessau Medical Center, Auenweg 38, Dessau, 06847, Germany; 2 Research Group Geriatrics, Charité Universitätsmedizin Berlin, Reinickendorfer Strasse 61, Berlin, 13347, Germany

**Keywords:** Malignant atrophic papulosis, Köhlmeier-Degos disease, Degos disease, Etiology, Pathophysiology, Clinical manifestations, Prognosis, Treatment, Review

## Abstract

**Definition of the disease:**

Malignant atrophic papulosis (MAP), described independently by Köhlmeier and Degos et al., is a rare, chronic, thrombo-obliterative vasculopathy characterized by papular skin lesions with central porcelain-white atrophy and surrounding teleangiectatic rim.

**Epidemiology:**

Less than 200 cases have been described in the literature. The first manifestation of MAP usually occurs between the 20th and 50th year of life.

**Clinical description:**

The cutaneous clinical picture is almost pathognomonic. The histology is not consistent but in most cases it shows a wedge-shaped connective tissue necrosis in the deep corium due to a thrombotic occlusion of the small arteries. In the systemic variant, manifestations mostly occur at the intestine and central nervous system.

**Etiology:**

The etiopathogenesis of the disease remains unknown, a genetic predisposition may occur. Vasculitis, coagulopathy or primary dysfunction of the endothelial cells have been implicated.

**Diagnostic methods:**

Diagnosis is only based on the characteristic skin lesions.

**Differrential diagnosis:**

It depends on the clinical presentation of MAP, but systemic lupus erythematosus and other connective tissue diseases need to be considered.

**Management:**

No effective treatment exists for the systemic manifestations, while compounds that facilitate blood perfusion have achieved a partial regression of the skin lesions in single cases.

**Prognosis:**

An apparently idiopathic, monosymptomatic, cutaneous, benign variant and a progressive, visceral one with approx. 50% lethality within 2–3 years have been reported. Systemic manifestations can develop years after the occurrence of skin lesions leading to bowel perforation and peritonitis, thrombosis of the cerebral arteries or massive intracerebral hemorrhage, meningitis, encephalitis, radiculopathy, myelitis.

## Introduction

The malignant atrophic papulosis (Köhlmeier-Degos disease; MAP) was described by Köhlmeier in 1941 [1] and documented as a separate entity by Degos et al. one year later [2]. Although MAP has been known for almost 70 years, its pathomechanism remains still obscure. As a result no treatment has been proven sufficient enough to cope with the disease.

It is a rare disease; until today less than 200 cases have been described in the literature. The first manifestation of MAP usually occurs between the 20th and 50th year of life [3,4], while single cases with MAP in newborns and children have also been described [5,6]. A genetic predisposition with an autosomal dominant trait has been suggested, since there has been reports about more frequently affected 1st degree relatives [7-9].

The following article presents an overview of MAP as well as a summary of the proposed theories of disease development. The exact knowledge of MAP research history may lead to new unexplored pathways and eventually to the discovery of the pathogenesis of this potentially lethal illness.

### Clinical manifestations

**Figure 1:**
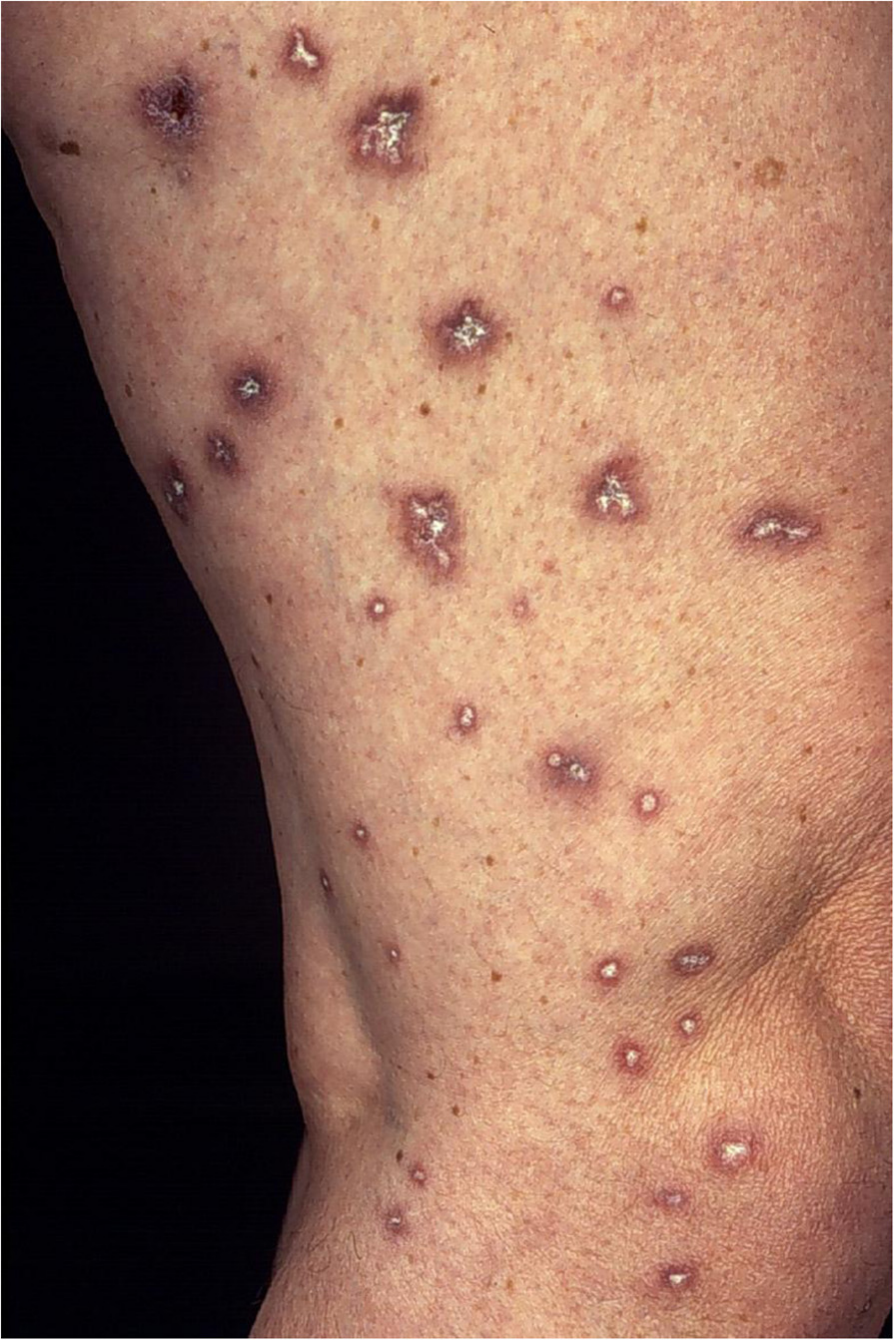
Cutaneous involvement of Köhlmeier-Degos disease showing scattered typical lesions on the lower extremities of a female patient.

**Figure 2:**
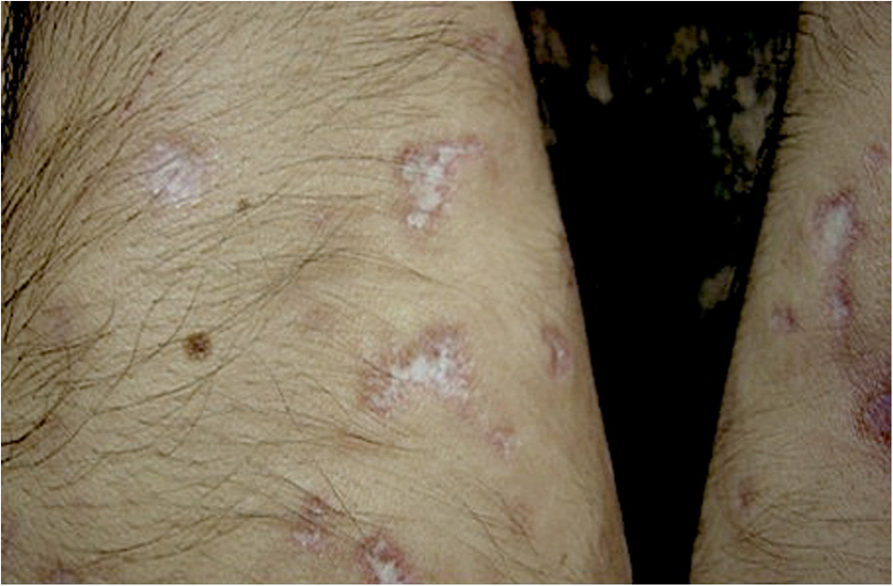
Characteristic lesions with porcelain-white center und a surrounding erythematous rim on the upper extremities of a male patient.

The diagnosis of MAP is based in the majority of the cases on the pathognomonic skin lesions. They are about 0.5-1 cm large papules with an atrophic porcelain-white centre and an erythematous, teleangiectatic rim mostly occurring on the trunk and the upper extremities [4,10] (Figures 1, 2). The lesions appear initially as small erythematous papules. After a few days the centre sinks and they start to demonstrate the characteristic morphology. Palms, soles, scalp and face are rarely involved. On the other hand, involvement of the internal organs, with multiple limited infarcts of the intestine and/or the central nervous system (CNS) as well as of other organs, such as the lungs (presenting as pleuritis and/or pericarditis) and the eyes, has also been reported [11-14].

### Prognosis

Due to the markedly different prognosis between the apparently idiopathic cutaneous disease and MAP with systemic involvement, the first variant - in contrast to the latter "malignant" one - has been termed "benign atrophic papulosis" by some authors [15,16]. However, it is still unclear if these two forms can be unambiguously distinguished from each other, since systemic involvement can develop year after the occurrence of the skin lesions.

The so called "benign" form of the disease is characterized by the typical skin lesions, which persist over years or lifelong, without involvement of the inner organs [17]. Several cases have exhibited signs of inheritance, especially between first-degree relatives [7,9]. The malignant variant is characterized by involvement of the skin and the inner organs, either occurring simultaneously or subsequently. The systemic manifestations can be followed in many cases by serious complications, namely bowel perforation and peritonitis as well as thrombosis of the cerebral arteries or massive cerebral hemorrhage, meningitis, encephalitis, radiculopathy, myelitis [18] leading to lethal course in approx. 50% of the patients within 2 to 3 years. Lung involvement can be followed by pleuritis and/or pericarditis [10,19,20]. The prognosis can also be influenced by the extent of these ischemic complications, which are the determinants of mortality [2,8,21]. An ocular involvement with affection of the eyelids, conjuctiva, retina, sclera and the choroid plexus, as well as the development of diplopia and ophthalmoplegia as secondary side effects of the neurologic involvement have also been described [22]. The fact that a systemic involvement can develop suddenly, years after the occurrence of skin lesions, makes a regular medical follow-up of the patients necessary.

### Diagnosis

**Figure 3:**
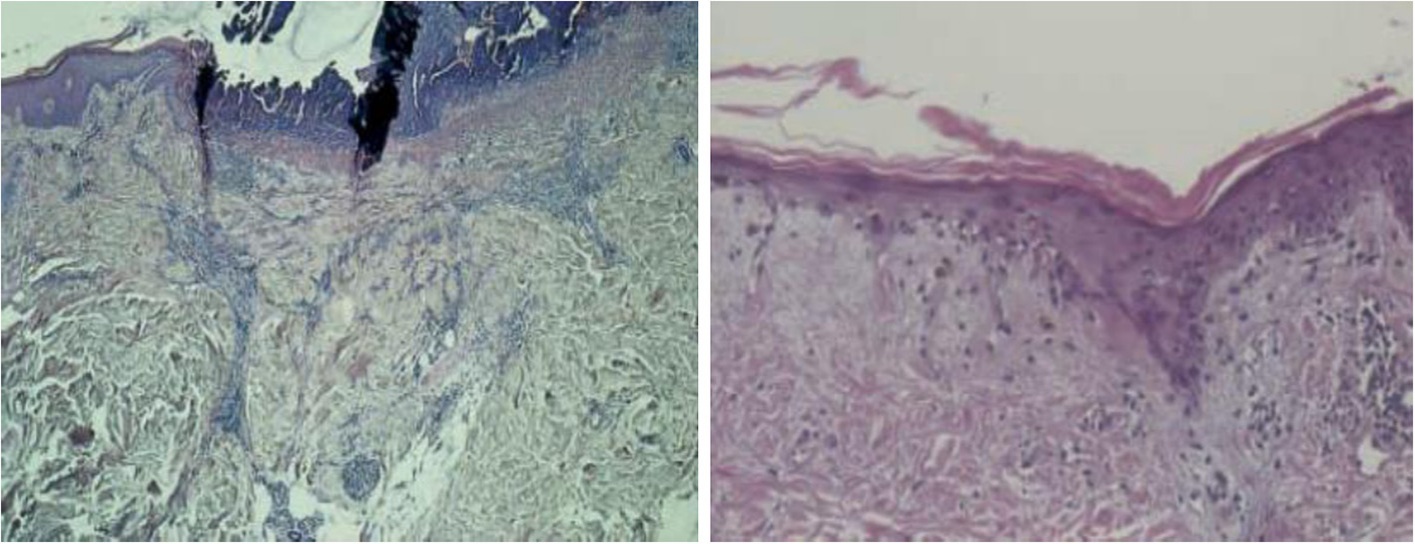
Biopsy from skin lesions showing a wedge-shaped necrosis, arteriolar obliteration, epidermal atrophy, hyperkeratosis and disarrangement of the collagen fibers in the corium.

The diagnosis of MAP is a clinical one and can be supported by the histological findings. The classical histology shows a wedge-shaped connective tissue necrosis, due to thrombotic occlusion of the small arteries deep in the corium [20,23,24]. However, these characteristic features cannot be demonstrated in all cases [25]. Harvell et al. [26] examined in a case report the histology of the lesions according to the duration of their existence. Early lesions have shown a superficial and deep perivascular lymphocytic infiltration, with distinct mucin deposition, which resembled lupus erythematosus. The fully developed lesions had more prominent changes in the dermoepidermal junction, with atrophy of the epidermis and an area of sclerosis in the papillary dermis. These characteristics could be compatible with a minimal variant of lichen sclerosus et atrophicans. The late lesions showed a wedge-shaped necrosis, sparse lymphocytes and markedly less mucin deposition in comparison to the early and fully developed lesions (Figure 3).

No specific alterations of laboratory parameters - if any - have been reported [10] and no markers exist, which could verify the diagnosis. However, a relative large percentage of the patients has been described to present defects of blood coagulation.

### Etiology and pathogenesis

The etiology of MAP remains unexplained. There is a whole series of hypotheses but none of them could be proven yet. The 3 most reasonable suggested hypotheses about the pathophysiology of the disease are vasculitis, coagulopathy and primary dysfunction of the endothelial cells [27]. Although heterogeneous the aforementioned etiological suggestions are not necessarily mutually exclusive. The simultaneous presence of various factors creating the appropriate conditions for the development of thrombosis should be considered.

#### Malignant atrophic papulosis as vasculitis

Soter et al. [24] have proposed that inflammation of the vessels could act as a trigger factor for the development of MAP. This inflammation was evaluated as an initial stadium of the disease, as in the histological samples of patients with MAP did not always demonstrate inflammatory cells. Su et al. [28] described a “lymphocyte-associated necrotic vasculitis” as the most prominent cutaneous feature of the skin lesions. In addition, they observed an analogy between the disseminated vasculitic process of the disease and the skin lesions of some patients with lupus erythematosus, which seemed similar. Currently, Magro et al. [29] have reported prominent C5b-9 deposits in skin, gastrointestinal tract and brain vessels of 4 patients with MAP, who have died from the disease. All cases had evidence of high expression of interferon-α (based on tissue expression of MXA, a type I interferon-inducible protein), endothelial tubuloreticular inclusions, and an interferon gene signature in peripheral blood mononuclear cells. The MXA expression paralleled the pattern of C5b-9 deposition.

#### Degos disease as coagulopathy

A thrombus deep in the dermis (stratum reticulare) is the primary event in MAP. The reduction of the blood flow and the resulting damage of the endothelial cells lead to deposition of mucin and aggregation of mononuclear cells [30]. Several authors have observed fibrinolytic dysfunction in selected patients [21,31-33]. Stahl et al. [34] and Drucker [35] have described single patients who showed an increased platelet aggregation in vivo. Both patients responded very well on the treatment with platelet aggregation inhibitors, namely aspirin and dipyridamole. Black et al. [36] observed a complete loss of fibrinolysis around the small blood vessels, in the centre of old and new papules in skin lesions of patients with MAP. Vazquez-Doval et al. [4] and Olmos et al. [18] described an increase of the activity of plasminogen activator inhibitor-1, while Paramo et al. [31] found that the serum level of plasminogen was decreased in a patient with MAP. Alternatively, Englert et al. [37], Mauad et al. [38] and Farell et al. [39] treated single patients with positive lupus anticoagulant. In addition to that, Yoshikawa et al. [40] have described a persistent increase of the thrombin-antithrombin III complex and of plasmin-α-2 plasmin inhibitor complex. All these observations may provide an explanation for the pathogenesis of MAP. Currently, Meephansan et al. [41] observed strong staining of the infiltrating inflammatory cells in the perivascular, intravascular, and perineural areas in tissue samples from 2 MAP patients with stromal cell–derived factor (SDF)-1/CXCL12, which is secreted by bone-marrow stromal and endothelial cells, activates megakaryocyte precursors, and costimulates platelet activation.

#### MAP as primary or secondary dysfunction of the endothelial cells

Tribble et al. [42] supposed that an abnormal swelling and proliferation of the vascular endothelium could trigger cutaneous, intestinal and central nervous system thrombosis. Howard and Nishida [43,44] observed tubulo-reticular aggregates in the endothelial cells with the help of electron microscopy. Therefore, a viral or bacterial infection could act as a cause for the endothelial changes [43-45]. Other authors showed intracytoplasmic paramyxovirus-like inclusions in electron microscopy of skin specimens from patients with MAP [18,35]. However, no proof of paramyxovirus-DNA in skin biopsies of patients has been provided via polymerase chain reaction [31].

### Management

There is no uniformly effective therapy for MAP. Efforts with fibrinolytic and immunosuppressive therapeutic regimens like cyclosporine A, azathioprine, cyclophosphamide and corticosteroids have been mostly unsuccessful. Furthermore, there have been reported cases where MAP worsened during immunosupression [7,46]. Explorative treatment with eculizumab could not prevent the development or progression of systemic manifestations (personal communications), despite its reported initial effectiveness on skin and intestinal lesions [47,48]. Other therapeutic efforts with anticoagulants and compounds that facilitate blood perfusion, such as acetylosalicylic acid (aspirin), pentoxifylline, dipyridamole, ticlodipine and heparin, have achieved a partial regression of the skin lesions in single cases [4,11,28,34,39,49,50]. Therefore, these agents can be used as a first therapeutic approach on a newly diagnosed patient with MAP. Subcutaneous treprostinil has currently been tested successfully in a case with eculizumab-resistant MAP with intestinal and CNS manifestations (Dr. Lee S. Shapiro, Albany, NY, personal communication).

Since every diagnosed MAP case can potentially develop into the systemic, life-threatening variant, an annual follow-up is mandatory. This should include a clinical inspection of the skin combined with additional examinations, such as brain magnetic resonance tomography, gastroscopy and colonoscopy, as well as X-ray of the chest and abdominal ultrasound in order to assess the long-term prognosis.

## Conclusion

MAP is a rare and potentially life-threatening disease. The prognosis highly depends on the development of systemic involvement. Most of the lethal courses are due to bowel perforation or CNS lesions and their consequences. The probability of a benign course increases with the duration of monosymptomatic cutaneous disease.

## Abbreviations

CNS: Central nervous system; MAP: Malignant atrophic papulosis.

## Competing interests

The authors declare that they have no competing interests.

## Authors’ contributions

AT wrote the manuscript. AT, EM and CCZ read, revised and approved the final manuscript. EM and CCZ share senior authorship.

## Authors’ information

The work is dedicated to Mrs. Judith Calder, Degos Patients' Network, who has been a long-term motivator and supporter for the initiation and continuation of research in this rare disease.
